# [^68^Ga]Ga-PSMA-11 PET before and after initial long-term androgen deprivation in patients with newly diagnosed prostate cancer: a retrospective single-center study

**DOI:** 10.1186/s13550-020-00723-0

**Published:** 2020-11-06

**Authors:** Sebastian Hoberück, Steffen Löck, Robert Winzer, Klaus Zöphel, Michael Froehner, Dieter Fedders, Jörg Kotzerke, Tobias Hölscher

**Affiliations:** 1 Department of Nuclear Medicine, Faculty of Medicine, University Hospital Carl Gustav Carus, TU Dresden, Fetscherstr. 74, 01307 Dresden, Germany; 2 OncoRay – National Center for Radiation Research in Oncology, Faculty of Medicine, University Hospital Carl Gustav Carus, Technische Universität Dresden, Helmholtz-Zentrum Dresden - Rossendorf, Dresden, Germany; 3 Department of Radiology, Faculty of Medicine, University Hospital Carl Gustav Carus, TU Dresden, Dresden, Germany; 4 Department of Nuclear Medicine, Klinikum Chemnitz gGmbH, Chemnitz, Germany; 5 Department of Urology, Faculty of Medicine, University Hospital Carl Gustav Carus, TU Dresden, Dresden, Germany; 6 Department of Urology, Zeisigwaldkliniken Bethanien Chemnitz, Chemnitz, Germany; 7 Department of Radiotherapy and Radiation Oncology, Faculty of Medicine, University Hospital Carl Gustav Carus, TU Dresden, Dresden, Germany

**Keywords:** PSMA, ^68^Ga, ADT, Prostate cancer, PET

## Abstract

**Purpose:**

The study aimed to evaluate the effect of androgen deprivation therapy (ADT) on PSMA imaging and its correlation to the PSA concentration by comparing qualitative and quantitative parameters: SUV_max_, SUV_mean_, PSMA-derived tumor volume (PSMA-TV), total lesion PSMA (TL-PSMA) and molecular imaging (mi)PSMA score.

**Methods:**

Retrospective analysis of 21 therapy-naïve patients with oligometastatic prostate cancer (median age 70 years) who underwent either [^68^Ga]Ga-PSMA-11-PET/CT or -PET/MRI before initiation of (T1) as well as during ADT (T2). The median duration of ADT was 155 days (range 61–289 days). All lesions were analyzed using several qualitative and quantitative PET parameters.

**Results:**

A total of 109 PSMA-positive lesions (24 intraprostatic, 56 lymphonodal and 29 osseous) were visually detected at any of the examinations, while at T2, two new bone lesions were detected in one patient. During ADT, all patients experienced a decrease in their PSA level (median: 29.1 before vs. 0.71 after; *p* < 0.001). During long-term ADT, a relevant decrease in lesion count occurred, especially in patients with a T2 PSA value < 1 ng/ml (median: 4 vs. 0.9; *p* = 0.007) and PSMA expression, which resulted in miN- and/or miM-downstaging in 11 patients (52.7%).

All analyzed PET parameters correlated strongly with each other. The PSA level at T2 correlated modestly with the decrease in PSMA expression and its derived volumes.

**Conclusion:**

Post-ADT scans detected, especially in patients with a residual PSA < 1 ng/ml, fewer PSMA-positive lesions with overall lower PSMA expression, regardless of primary tumor site or metastatic sites. None of the PET parameters has proven to be superior, as they all correlated modestly with the PSA value at T2. Thus, the simply acquirable miPSMA score seems to be the most suitable for evaluating the effect of ADT on PSMA expression.

## Introduction

Prostate cancer (PCa) is the world’s most common cancer in men [1]. [^68^Ga]Ga-labeled PSMA ligands have become state of the art in molecular imaging of PCa in primary and recurrent diseases as well as in therapy monitoring [2–6].

In high-risk or oligometastatic situations, therapy includes androgen deprivation therapy (ADT) and radiation therapy [7, 8]. Recently, docetaxel chemotherapy or enhanced ADT (i.e., abiraterone, enzalutamide or apalutamide) has been recommended in castration-sensitive metastatic prostate cancer [9–13].

As PSMA-based imaging becomes increasingly important for planning local ablative therapy, the influence of ADT on PSMA expression is of high relevance [14].

On a cellular level, activated androgen receptors downregulate folate hydrolase 1 (FOLH1) gene expression. As the PSMA promoter and PSMA enhancer are located within FOLH1, its downregulation results in decreased PSMA expression. ADT reduces androgen receptor activation and reverses FOLH1 downregulation, leading to higher PSMA expression [15].

The influence of ADT on PSMA expression has been evaluated in several studies that showed the conflicting results. In a preclinical study, Murga et al. [16] showed PSMA upregulation in both androgen-sensitive and androgen-resistant prostate cancer cells. In an animal study, Evans et al. [17] reported a decreasing cell count under ADT and a higher PSMA expression per cell. These findings may affect imaging: the effect of increased PSMA expression in surviving cells may be overcompensated for by induced cell death in the vast majority of cells [14].

In the present study, the influence of ADT on PSMA expression in the primary tumor as well as in lymphatic and osseous metastases in exclusively untreated, hormonally naïve, oligo-metastasized patients was evaluated.

As surrogate parameters for PSMA expression, we compared the change in SUV_max_, SUV_mean_, PSMA-derived tumor volume (PSMA-TV) and total lesion PSMA (TL-PSMA) [18], and the miPSMA score [19] under ADT was compared for the primary tumor as well as the lymphatic and bone metastases within each other and the respective PSA values.

## Material and methods

### Patients

**Fig. 1:**
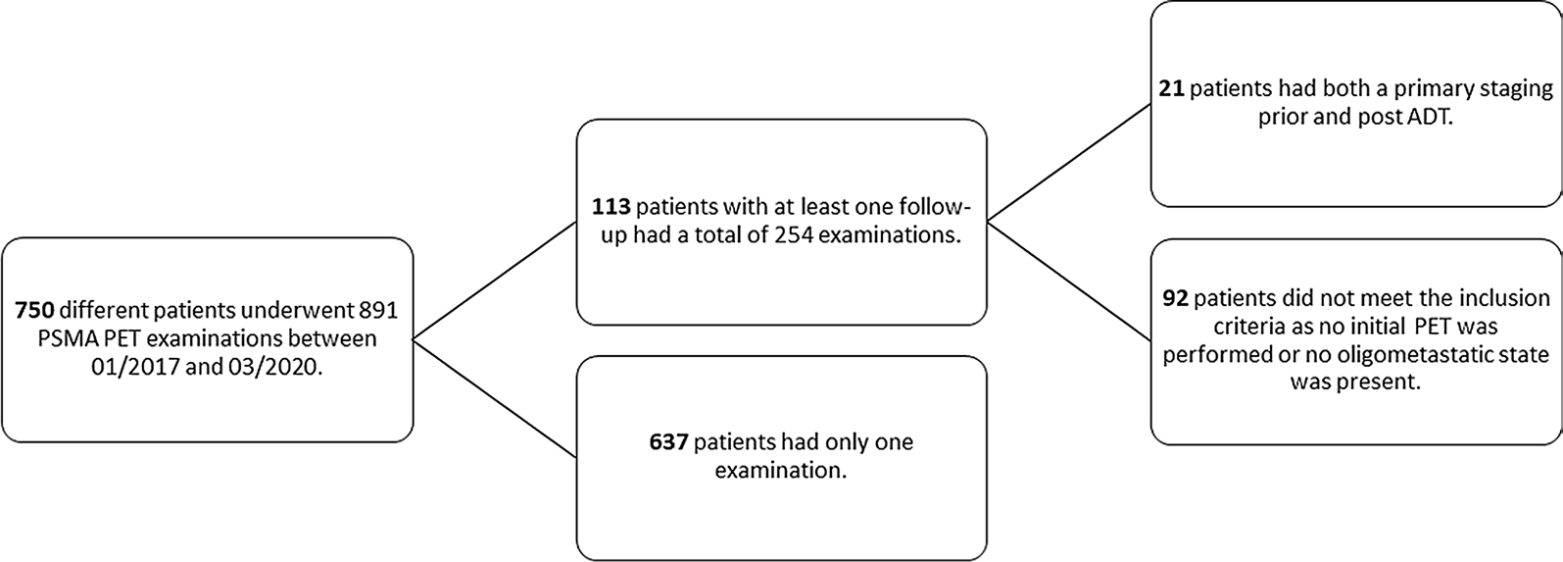
Patients referred for [^68^Ga]Ga-PSMA-11 PET to our department between 01/2017 and 03/2020

Twenty-one therapy-naïve, oligo-metastasized (up to 5 extraprostatic and extrapelvic lesions), biopsy-proven patients with prostate cancer with a median age of 70 years (range 57–80 years) who were foreseen for local ablative radiotherapy underwent [^68^Ga]Ga-PSMA-11-PET for primary staging before the start of androgen deprivation therapy (median: 14, range 0–59 days). All patients in this retrospective single-center study were discussed by members of an interdisciplinary tumor board. After a median of six months (range 61–289 days after the start of ADT), a restaging PET was performed, and local ablative radiotherapy to all known lesions was planned. The two time points were labeled T1 and T2. The data of all PSMAPET examinations performed in our department between 11/2016 and 03/2020 (Fig. 1) were obtained and were retrospectively analyzed.

Written informed consent was obtained from all patients for the clinically indicated examination and the consecutive scientific analysis of their clinical and imaging data. The institutional review board of the local ethics committee at our medical faculty approved this analysis.

### Radiotracer preparation

The radiotracer [^68^Ga]Ga-PSMA-11 was synthesized as in clinical routine and as previously described [20].

### Imaging protocol

No specific patient preparations were required for [^68^Ga]Ga-PSMA-11 PET.

For the 21 examinations at T1, a median of 153 MBq (range 90–206 MBq) was applied, and acquisition started with a median of 119 min p.i. (range 89–168 min), while for the examinations at T2, a nearly equal median of 155 MBq (range 62–190 MBq) was applied, and imaging started with a median of 116 min p.i. (range 92–140 min).

The 42 examinations were performed on either PET/MRI or PET/CT 1 or PET/CT 2. Ten out of the 19 patients underwent both examinations on the same device.

The PET/CT scans until 08/2019 (PET/CT 1) were acquired with a Biograph 16 (Siemens CTI, Knoxville, Tennessee, USA). Eight to nine bed positions were obtained with a 3-min scan time each. The PET/CT scans after 08/2019 (PET/CT 2) were acquired with a Biograph Vision 600 (Siemens Healthineers, Knoxville, USA). The emission PET scan was obtained using continuous bed motion with a speed of 2.9 mm/s being equivalent to 1.5 min per bed position.

The PET/MRI scans were acquired with a 3 T Ingenuity TOF PET/MR (Philips Medical Systems, Best, the Netherlands). Ten bed positions were acquired with a scan time of 3 min each.

### Imaging reconstruction

The CT 1 images were reconstructed using an ordered subset expectation maximization (OSEM) algorithm with 6 iterations and 4 subsets with a 168 × 168 matrix. Plain CT scans for attenuation correction were performed in a craniocaudal direction from the skull base to the upper thighs. Scanning parameters included 100 mA s, 120 kV, online tube current modulation, 1.5-mm slice collimation, 0.5–0.75-s rotation time and reconstruction of 5-mm slices.

The CT 2 images were reconstructed using the TRueX algorithm with 4 iterations, 5 subsets, time-of-flight (TOF) application and without filtering. The resulting PET images had an image matrix size of 440 × 440 with a voxel size of 1.65 × 1.65 × 3.0 mm. A standard low-dose CT was acquired from the whole body (X‐ray tube current of 10 mAs, tube voltage of 100 kV, spiral pitch factor of 1.5 and 3.0-mm slice thickness) and used for scatter correction of the subsequent PET scan.

For a lesion-based assessment, the different quantitative parameters were obtained, and correction with the SUV_mean_ of the liver was performed to minimize the effect of different reconstruction algorithms in the three different devices, as no further homogenization in the reconstruction algorithms could be performed.

### Image analysis

A nuclear medicine physician (SH) and a radiologist (RW), both experienced in PSMA PET reporting, used Syngo.via Software (VB30a, Siemens Healthineers, Erlangen, Germany) to determine pathological uptakes and to identify the reference lesions. Senior consultants in nuclear medicine (KZö) and radiology (DF) retrospectively confirmed the findings of both of them.

First, all scans were evaluated visually. Pathological uptakes were initially assumed if a lesion showed a tracer uptake higher than the local background [21]. Depending on the localization, they were rated as local (prostate) tumor, lymphonodal or bone metastasis. For subsequent quantitative analysis, volumes of interests (VOIs) sufficiently large for covering the whole lesion were inserted over each pathological lesion, and the SUV_max_ and SUV_mean_ of each lesion were acquired. The resulting volumetric parameters were the PSMA-derived tumor volume (PSMA-TV) based on a 45% cutoff of the SUV_max_, as suggested by Schmuck et al. [18], and the total lesion PSMA (TL-PSMA), which is a product of PSMA-TV and the SUV_mean_ of that lesion. The concept of these molecular volumes is adapted from FDG imaging, and PSMA-TV calculation is equivalent to the molecular tumor volume (MTV), while TL-PSMA is calculated equally to the total lesion glycolysis (TLG) [22].

Sufficiently large [19] VOIs were further inserted in reference regions: liver (3-cm diameter), thoracic aorta (2-cm diameter) and parotid glands (1.5-cm diameter), and the SUV_max_ and SUV_mean_ values were calculated. For the parotid glands, the values were averaged.

To make the uptake values more comparable between the different devices and different reconstruction algorithms, ratios to the respective liver SUV_mean_ were calculated (LQ) for SUV_max_, SUV_mean_ and TL-PSMA and compared.

For the same reason, each lesion was scored according to the miPSMA expression score (19). The score ranges from 0 (uptake < blood pool) to 3 (uptake ≥ parotid gland). It was determined based on the SUV_mean_ of both the lesions and the reference lesions. If a lesion was not separable from the local background at one time point, it was scored as 0, regardless of its SUV_mean_. To evaluate the patients’ total tumor burden, the sum of the scores of all lesions was calculated as well.

Furthermore, each patient was staged using the miTNM expression score. Since there was no contrast-enhanced CT or MRI simultaneously acquired after ADT, there was no T-stage to be compared.

### Statistical analysis

To compare different PSMA parameter lesion-based characteristics and the PSA value between the two time points T1 and T2, the paired Wilcoxon signed-rank test was applied. For the comparison of parameters between independent patient groups or lesions, the Mann–Whitney *U* test was used. Correlations between PSMA parameters, lesion-based characteristics and PSA values were evaluated by the Spearman correlation coefficient r. All statistical analyses were performed using SPSS 25 (IBM Corporation, Armonk, NY, USA). Two-sided tests were performed, and *p* values below 0.05 were considered statistically significant.

## Results

**Fig. 2:**
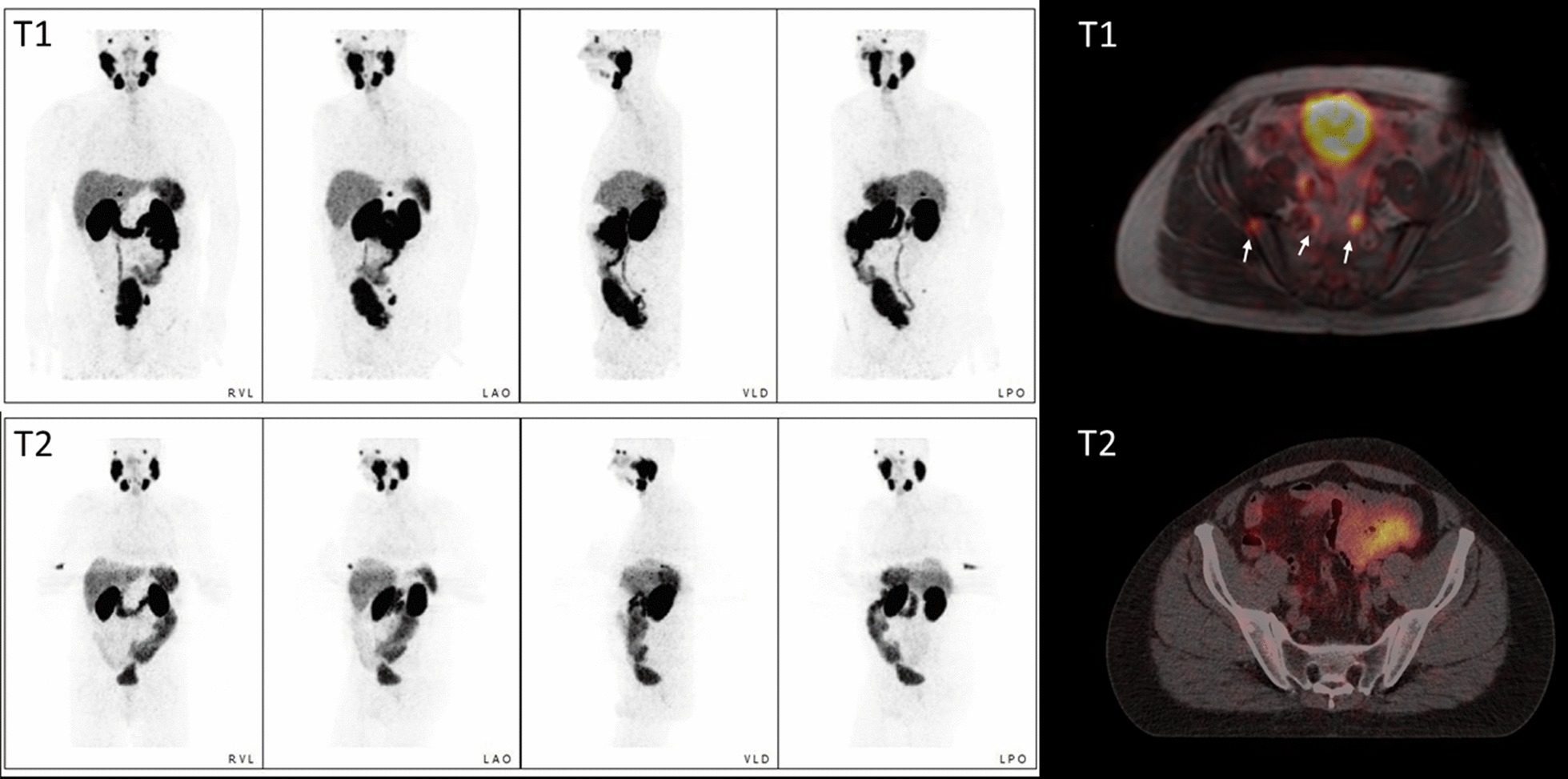
MIPs and fused PSMA PET/CT of Patient #3. The MIPs show a clearer demarcation of the prostatic tumor as well as the complete regression of lymphonodal metastases, whereas two new osseous metastases occurred (the lower one is indicated by the blue arrow)

As shown in Table 1, 21 patients received ADT for a median of 155 days (range 61–289 days) prior to local ablative radiotherapy. Meanwhile, the median PSA value dropped from a median value of 29.1 ng/ml (range 2.5–107.0 ng/ml) to 0.71 (0.05–4.91) ng/ml (*p* < 0.001). Accordingly, the number of both PSMA-expressing intraprostatic and extraprostatic tumor manifestations dropped (107 at T1 vs. 50 [40.7%] at T2). The T2 lesions later included two new bone metastases that occurred in the same patient (Fig. 2), resulting in 109 lesions to analyze. In total, the tumor burden decreased in both number and size from T1 to T2. In total, the summed PSMA-derived tumor volume (PSMA-TV) was only 24.0% of the initial value, and the total lesion PSMA decreased even more (13.9% of T1).

**Table 1:** Patient characteristics

Characteristics	Results
Age [years], **median**, mean, range	**70**, 69, 57–80
Time between ADT initiation and second [^68^Ga]Ga-PSMA-PET [days], **median**, mean, range	**155**, 158, 61–289
PSA value at [^68^Ga]Ga-PSMA-PET (T1), **median**, mean, range [ng/ml]	**29.1**, 39.0, 2.5–107.0
PSA value at [^68^Ga]Ga-PSMA-PET (T2), **median**, mean, range [ng/ml]	**0.71**, 1.24, 0.05–4.91
Lesions T1 versus T2	
Prostate tumor [*n*: patients; *n*: lesions]	21 versus 17; 24 versus 18
Lymph node metastases [*n*: patients; *n*: lesions]	16 versus 10; 56 versus 20
Bone metastases [*n*: patients; *n*: lesions]	14 versus 10; 27 versus 12^a^
Sum [*n*: lesions]	107 versus 50^a^
**∑** PSMA derived tumor volume (PSMA-TV) of all lesions at T1, **sum** [ml]	**505.69**
**∑** PSMA derived tumor volume (PSMA-TV) of all lesions T2, **sum** [ml], (% of T1)	**121.51** (24.0%)
**∑** Total lesion PSMA (TL-PSMA) of all lesions at T1, **sum** [ml]	**9016.32**
**∑** Total lesion PSMA (TL-PSMA) of all lesions at T2, sum [ml], (% of T1)	**1256.91** (13.9%)

^a^Two of these were new in T2

Table 2 provides further details of the patients’ disease, ADT duration and treatment history as decided by the interdisciplinary tumor board. Five patients received additional chemotherapy (CTx, docetaxel 75 mg/m^2^ q3wk). In 9 patients, the PSA concentration and ADT remained > 1 ng/ml (bad responders). These patients, however, had a significantly shorter therapy duration (mean: 114 days vs. 119 days; p: 0.002), while having no differences in initial PSA (39.7 vs. 38.4 ng/ml; p: 0.925).

**Table 2:** Patient-based characteristics

Pat	Age	T1	T2	GSC	ADT	ADT (days)	CTx	Initial PSA (ng/ml)	PSA after ADT		∑ PSMA-TV at T2 in % of T1	∑ TL-PSMA at T2 in % of T1
									(ng/ml)	[% of T1]		
1	60	MR	CT2	4 + 3	B	219	6 Cycles	80	0.41	0.5	4.50	5.89
2	64	CT1	CT2	5 + 4	L	289	6 Cycles	5.9	0.15	2.5	2.80	4.34
3	59	CT1	CT1	5 + 4	Max	279	6 Cycles	2.49	0.27	10.8	12.62	8.95
4	74	CT1	CT2	4 + 4	Max	149	X	29.14	0.69	2.4	9.65	15.78
5	57	MR	CT2	4 + 3	Max	218	X	45.4	0.71	1.6	38.12	94.63
*6*	*74*	*MR*	*MR*	*4* + *4*	*Max*	*61*	*X*	*57.5*	*1.42*	*2.5*	*35.12*	*28.80*
7	73	MR	MR	4 + 5	Max	98	X	14.06	0.1	0.7	16.71	11.42
8	74	CT1	MR	4 + 3	Max	115	X	25.8	0.05^a^	0.2	23.35	2.94
*9*	*79*	*MR*	*CT1*	*4* + *5*	*Max*	*156*	*X*	*42.06*	*1.9*	*4.5*	*44.38*	*33.59*
*10*	*79*	*MR*	*CT1*	*4* + *4*	*B*	*116*	*X*	*17.97*	*1.52*	*8.5*	*53.13*	*50.02*
*11*	*76*	*CT1*	*CT1*	*4* + *3*	*Max*	*118*	*X*	*100.4*	*1.93*	*1.9*	*39.57*	*9.29*
12	65	MR	CT1	4 + 5	Max	155	X	91.5	0.09	0.1	0.00	0.00
*13*	*59*	*CT1*	*CT1*	*4* + *4*	*Max*	*81*	*X*	*32.9*	*3.08*	*9.4*	*206.69*	*267.85*
14	66	CT1	CT1	4 + 4	Max	168	6 Cycles	107	0.21	0.2	42.53	6.50
15	74	CT1	CT1	4 + 4	Max	174	X	16.17	0.93	5.8	16.96	2.73
16	80	CT1	CT1	4 + 5	Max	253	X	12.07	0.45	3.7	63.50	30.34
17	66	CT1	CT1	3 + 4	Max	205	4 Cycles	31.5	0.05	0.2	0.00	0.00
*18*	*70*	*CT1*	*CT1*	*4* + *4*	*Max*	*109*	*X*	*21*	*4.91*	*23.4*	*59.97*	*51.12*
*19*	*81*	*CT2*	*CT2*	*4* + *3*	*Max*	*102*	*X*	*26.13*	*3.53*	*13.5*	*45.78*	*40.14*
*20*	*57*	*CT2*	*MR*	*5* + *4*	*Max*	*134*	*X*	*36.2*	*1.34*	*3.7*	*52.09*	*6.18*
*21*	*59*	*CT2*	*CT2*	*4* + *3*	*Max*	*158*	*X*	*23.5*	*2.26*	*9.6*	*93.89*	*90.35*

GSC, Gleason score; ADT, androgen deprivation therapy; CTx, chemotherapy/in all cases Docetaxel; B, bicalutamide 150 mg/d; L, leuprorelin 11.25 mg/3 months; Max, maximal androgen blockade (leuprorelin 11.25 mg/3 months + bicalutamide 50 mg/d)

Changes in PSA and PSMA-derived tumor volumes (primary tumor, lymph node metastases and bone metastases), PSMA-TV and TL-PSMA. Patients with a T2 PSA > 1 ng are in italics

^a^Patient 8 underwent TUR-P in the meantime

**Fig. 3:**
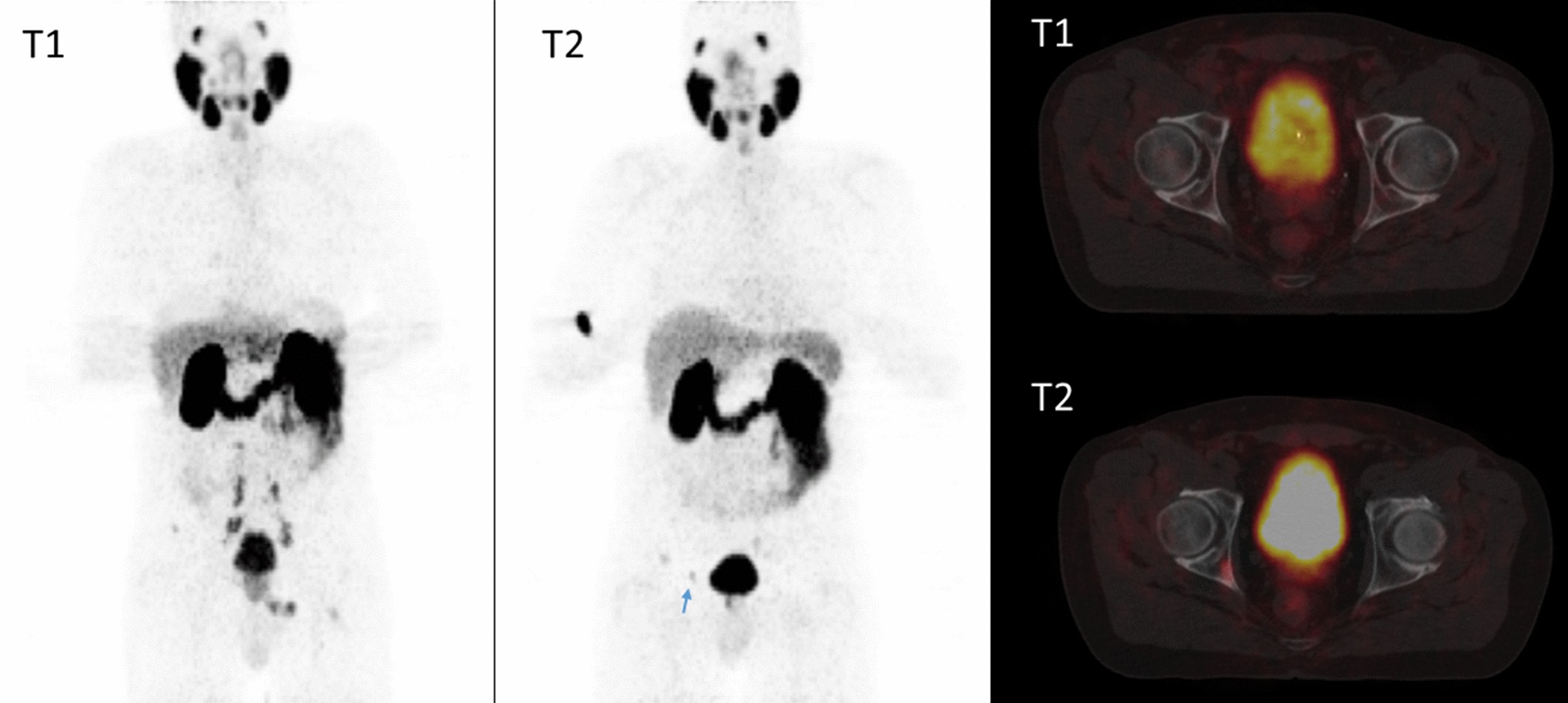
MIPs and fused PET/MRI at T1 and PET/CT at T2. There is no pathological PSMA expression in either the prostate or the metastatic sites (arrows in the fused image at T1) at T2

While two patients with a good PSA response showed complete remission under ADT, as exemplarily shown in Fig. 3, one patient (No. 13) with an insufficient PSA response presented with a doubling of his metabolic tumor volumes in the prostate and solitary bone metastasis.

Table 3 outlines that a relevant portion of the patients had a post-ADT decrease in lesion counts (*n* = 16; 76.2%) and PSMA expression as shown by the summed miPSMA score (*n* = 20; 95.2%) and in the resulting miN-stage (*n* = 7; 33.3%) and miM-stage (*n* = 7; 33.3%). A combined change in miNM-stage occurred in 11 patients (52.4%). As the T-stage could not reliably be evaluated in native imaging, the miT-stage was not reevaluated.

**Table 3:** Patient-based analysis of detectable lesions prior (T1) and during ADT (T2)

Pat	Prostatic lesions [*n*]		Lymph node metastases [*n*]		Bone metastases [*n*]		T-stage	miN-stage		miM-stage		Max_Score		Summed score	
	T1	T2	T1	T2	T1	T2	T1/T2	T1	T2	T1	T2	T1	T2	T1	T2
1	1	1	7	2	3	2	T3	N1b	N1b	M1b	M1b	3	2	23	6
2	2	1	4	1	4	1	T1	N1b	*N1a*	M1b	M1b	3	3	21	7
3	1	1	7	0	5	2^a^	T3	N1b	*N0*	M1b	M1b	2	2	21	4
4	1	1	10	7	1	0	T3	N1b	N1b	M1b	*M1a*	3	3	27	18
5	1	1	1	1	2	1	T2	N1a	N1a	M1b	M1b	2	2	5	4
6	1	1	2	1	1	1	T2	N1b	*N1a*	M1b	M1b	2	3	8	6
7	1	1	6	0	0	0	T3	N1b	*N0*	M1a	*M0*	3	2	15	2
8	1	0	0	0	3	1	T3	N0	N0	M1b	M1b	2	0	6	0
9	1	1	1	0	0	0	T1	N0	N0	M1a	*M0*	2	1	3	1
10	1	1	0	0	1	1	T3	N0	N0	M1b	M1b	2	2	4	4
11	1	1	5	3	0	0	T3	N1b	N1b	M0	M0	3	2	16	6
12	1	0	1	0	2	0	T4	N1a	*N0*	M1b	*M0*	3	0	8	0
13	1	1	0	0	1	1	T1	N0	N0	M1b	M1b	2	2	4	3
14	1	1	1	1	1	0	T3	N1a	N1a	M1b	*M0*	2	1	6	2
15	1	1	1	0	0	0	T3	N1a	*N0*	M0	M0	3	2	5	2
16	1	1	0	0	1	1	T2	N0	N0	M1b	M1b	2	2	4	3
17	1	0	5	0	0	0	T1	N1b	*N0*	M1a	*M0*	2	0	8	0
18	1	1	3	2	1	0	T3	N1b	N1b	M1b	*M0*	3	3	8	5
19	2	0	0	0	1	1	T3	N0	N0	M1b	M1b	3	1	6	1
20	1	1	1	1	0	0	T3	N1a	N1a	M0	M0	3	2	5	3
21	2	2	1	1	0	0	T3	N1a	N1a	M0	M0	3	3	9	7
∑	24	18	56	20	27	12						53	38	212	84

^a^New manifestation under ADT

A separate analysis between patients with a T2 PSA value < 1 ng/ml and those with higher serum PSA levels (Table 4) showed significant differences between both groups. While patients with a residual PSA value < 1 ng/ml had 49 preknown lesions that were not retrievable at T2 (mean: 4.1), the patients with insufficient PSA response had only 8 (mean: − 0.9; *p* = 0.007). The remnant summed miPSMA score at T2 was far lower in patients with PSA < 1 ng/ml at T2 (29.7% vs. 62.5%; p: 0.036). Consecutively, patients with a low PSA value had not only fewer persistent lesions but also a lower residual molecular tumor volume (PSMA-TV) after ADT (median: 16.7% vs. 52%; *p*: 0.008). Even though the difference in total lesion PSMA failed the significance threshold, the same tendency became apparent. However, patients with a residual PSA > 1 ng/ml had a significantly shorter therapy duration (*p* = 0.007).

**Table 4:** Comparison between patients with a residual PSA value at T2 < 1 ng/ml and > 1 ng/ml

**–**	Patients with PSA < 1 ng/ml at T2 (*n* = 12)	Patients with PSA > 1 ng/ml at T2 (*n* = 9)	All patients (*n* = 21)	*p* value
Irretrievable lesions at T2 [*n*]: sum; mean	49; 4.1	8; 0.9	57; 2.7	**0.007**
Irretrievable N and M [*n*]: sum; mean	45; 3.8	6; 0.67	51	**0.007**
T2/T1 of PSMA-TV [%]: median (range)	16.7 (0.0–63.5)	52.0 (35.1–207)	38.1 (0.0–207)	**0.008**
T2/T1 of TL-PSMA [%]: median (range)	6.2 (0.0–94.6)	40.1 (6.0–268)	11.4 (0.0–268)	0.062
T2/T1 of summed miPSMA score [%]: median (range)	29.7 (0.0–80.0)	62.5 (16.7–100)	37.5 (0.00–100)	**0.036**
Downstaged patients at T2: sum; [%]	8 [66.7%]	3 [33.3%]	11 [52.4%]	0.198
Duration of ADT [days], median, range	190 (98–289)	116 (61–162)	155 (61–289)	**0.002**
PSA at T1	27.5 (2.5–107.0)	32.9 (18.0–100.4)	29.1 (107.0)	0.925

*p* values < 0.05 were indicated bold

In the primary staging (T1), no correlation between the individual tumor burden and the pretherapeutic PSA concentration could be shown (Table 5). Under ADT (T2), the remaining total molecular tumor volume (TL-PSMA) in total (*r* = 0.504; *p* = 0.020) and intraprostatic tumor volume (*r* = 0.456; *p* = 0.038) correlated modestly with the PSA value at T2. The strongest correlations were observed between the PSA value after ADT (T2) and the T2/T1-quotients of the PSMA-derived tumor volumes. The simple sum of the miPSMA score of all lesions correlates only slightly worse with the corresponding PSA values than the far more elaborated PSMA-TV and TL-PSMA molecular volumes.

**Table 5:** Patient-based correlation between PSMA-derived tumor volumes and PSA values at different time points

All patients (*n* = 21)		miPSMA score ∑All lesions	PSMA_TV ∑All lesions	TL_PSMA ∑All lesions	PSMA_TV ∑Prostate	TL_PSMA ∑Prostate	PSMA_TV ∑Lymph nodes	TL_PSMA ∑Lymph nodes	PSMA_TV ∑Bone	TL_PSMA ∑Bone
	Parameter time point	T2	T2	T2	T2	T2	T2	T2	T2	T2
T2 PSA	Correlation coefficient	0.343	0.315	0.504*	0.392	0.456*	0.213	0.244	0.037	0.137

	Parameter time point	T2/T1	T2/T1	T2/T1	T2/T1	T2/T1	T2/T1	T2/T1	T2/T1	T2/T1
T2 PSA	Correlation coefficient	0.604*	0.753**	0.752**	0.564*	0.529*	0.572*	0.548*	0.304	0.513
T2/T1 PSA	Correlation coefficient	0.464*	0.615*	0.620*	0.436*	0.478*	**0.169**	0.217	0.075	0.304

^*^p < 0.05; **p < 0.001. All significant correlation coefficients are indicated bold

Fewer and lower correlations emerged between the PSA decrease (T2/T1) and the reduced tumor volumes at T2, suggesting that the influence of the initial (T1) PSA value can be neglected in favor of the post-ADT PSA value.

Similar to the patient-based evaluation, the lesion-based analysis revealed modest correlations between the PSA value after ADT (T2) and all of the PSMA parameters at T2 and their quotient T2/T1. Further details for both time points are listed in Table 6.

**Table 6:** Lesion-based correlation of PSA values and PSMA parameters at different time points

All lesions (*n* = 109)		Score	SUV_max_	SUV_max_-LN	SUV_mean_	SUV_mean_ -LN	PSMA-TV	TL-PSMA	TL-PSMA-LN
	Parameter time point	T1	T1	T1	T1	T1	T1	T1	T1
T1 PSA	Correlation coefficient	0.172	0.243*	0.233*	0.264*	0.247*	− 0.103	0.051	0.038

Parameter time point		T2	T2	T2	T2	T2	T2	T2	T2
T2 PSA	Correlation coefficient	0.463**	0.460**	0.453**	0.466**	0.461**	0.424**	0.442**	0.435**

Parameter time point		T2/T1	T2/T1	T2/T1	T2/T1	T2/T1	T2/T1	T2/T1	T2/T1
T2/T1 PSA	Correlation coefficient	0.185	0.167	0.175	0.177	0.186	0.154	0.197*	0.202*
T2 PSA	Correlation coefficient	0.472**	0.464**	0.478**	0.469**	0.478**	0.501**	0.520**	0.528**

LN: Intraindividual liver correction [value divided by hepatic SUV_mean_]

^*^p < 0.05; **p < 0.001. All significant correlation coefficients are indicated bold

Table 7 displays the decline in PSMA expression at T2 in prostatic, lymphatic and osseous manifestations, regardless of what SUV parameter was analyzed. For example, the average SUV_max_ of all lesions dropped from 20.44 prior to ADT to 8.35 (40.9%), and the miPSMA score dropped to the same extent (39.7%) after initiation of ADT.

**Table 7:** Lesion-based quantification parameters in T1 and T2

Parameter	Prostatic lesions T1 [mean; **CI**]	Prostatic lesions T2 [mean; **CI**]	*p*	Lymphonodal lesions T1 [mean; **CI**]	Lymphonodal lesions T2 [mean; **CI**]	*p*	Bone lesions T1 [mean; **CI**]	Bone lesions T2 [mean; **CI**]	*p*	All lesions T1 [mean; **CI**]	All lesions T2 [mean; **CI**]	*p*
Score	**2.33**; 2.06–2.60	**1.50**; 1.07–1.93	0.001	**2.00**; 1.82–2.18	**0.61**; 0.35–0.86	< 0.001	**1.52**; 1.26–1.78	**0.48**; 0.22–0.74	< 0.001	**1.94**; 1.81–2.08	**0.77**;0.58–0.96	< 0.001
SUV_max_	**29.13**; 20.11–38.15	**14.67**; 9.02–20.32	0.002	**20.65**; 15.50–25.81	**8.17**; 2.79–13.55	< 0.001	**12.84**; 8.27–17.41	**3.48**; 1.30–5.65	< 0.001	**20.44**; 16.86–24.02	**8.35**; 5.24–11.46	< 0.001
SUV_mean_	**17.44**; 11.99–22.89	**8.63**; 5.36–11.90	0.003	**13.48**; 10.09–16.86	**5.10**; 1.80–8.41	< 0.001	**8.36**; 5.37–11.36	**2.20**; 0.80–3.61	< 0.001	**12.99**; 10.72–15.26	**5.11**; 3.22–7.00	< 0.001
PSMA-TV [ml]	**11.82**; 8.13–15.51	**3.32;** 1.52–5.13	< 0.001	**2.99**; 1.39–4.60	**0.23**; 0.08–0.39	< 0.001	**1.88**; 1.24–2.52	**0.98**; 0.23–1.75	0.013	**4.64**; 3.29–5.98	**1.12**; 0.63 -1.60	< 0.001
TL-PSMA [ml]	**253.16**; 104.63–401.70	**33.95**; 9.68–58.22	< 0.001	**41.62**; 19.70–63.53	**4.62**; 0–10.48	< 0.001	**21.03**; 4.49–34.57	**6.33**; 0–13.65	< 0.001	**82.72**; 45.49–119.94	**11.53**; 4.99–18.07	< 0.001

The miPSMA score as a simple visually obtainable surrogate parameter for the decrease in PSMA expression during therapy correlates very strongly with the quantitative PET parameters SUV_max_, SUV_mean_ and their derived tumor volumes, regardless of the additional intraindividual correction with liver uptake. As shown in detail in Table 8, these strong correlations exist in all tumor sites, with the lowest values in the primary tumor site.

**Table 8:** Correlation between the miPSMA expression score and the other obtained metabolic parameters

T2/T1-quotient (Q) of miPSMA score	Q SUV_max_	Q LN_SUV_max_	Q SUV_mean_	Q LN_SUV_mean_	Q PSMA-TV	Q TL-PSMA	Q LN-TL-PSMA	Q PSA	T2 PSA
*All lesions*									
Correlation coefficient	**0.970**	**0.969**	**0.970**	**0.969**	**0.828**	**0.903**	**0.896**	0.185	**0.472**
*p* value	** < 0.001**	** < 0.001**	** < 0.001**	** < 0.001**	** < 0.001**	** < 0.001**	** < 0.001**	0.057	** < 0.001**
*Primary tumor*									
Correlation coefficient	**0.874**	**0.893**	**0.866**	**0.885**	**0.505**	**0.669**	**0.666**	0.257	0.162
*p* value	** < 0.001**	** < 0.001**	** < 0.001**	** < 0.001**	**0.012**	** < 0.001**	** < 0.001**	0.226	0.449
*Lymph node metastases*									
Correlation coefficient	**0.968**	**0.976**	**0.967**	**0.975**	**0.886**	**0.933**	**0.931**	0.105	**0.484**
*p* value	** < 0.001**	** < 0.001**	** < 0.001**	** < 0.001**	** < 0.001**	** < 0.001**	** < 0.001**	0.442	** < 0.001**
*Bone metastases*									
Correlation coefficient	**0.945**	**0.951**	**0.945**	**0.951**	**0.873**	**0.917**	**0.917**	0.132	**0.547**
*p* value	** < 0.001**	** < 0.001**	** < 0.001**	** < 0.001**	** < 0.001**	** < 0.001**	** < 0.001**	0.510	**0.003**

Q, quotient of T2/T1; LN, liver normalization [value divided by hepatic SUV_mean_]. Significant correclation coefficents and their exact* p*-value are marked bold

In addition to the decrease in PSMA expression in the vast majority of the lesions, there is a small number of lesions showing higher uptake values under ADT (5.5% if assessed by Score and 13.8% if assessed by SUV_max_). Table 9 outlines that the molecular volumes as well as the miPSMA score were decreased in more lesions at T2 compared to the SUV parameters. Intraindividual liver correction did not change the results. The SUV_max_ (*p* = 0.687) and SUV_mean_ (*p* = 0.453) of the three reference regions of the mediastinal blood pool, liver and salivary glands did not differ between both time points.

**Table 9:** Lesion-based comparison of the values at T2 and T1

	Value in T2 ≤ T1							
	Score	SUV_max_	SUV_max__LN	SUV_mean_	SUV_mean__LN	PSMA-TV	TL-PSMA	TL-PSMA-LN
Prostatic lesions [*n* = 24]	23/24	20/24	19/24	19/24	17/24	23/24	22/24	22/24
Lymphonodal lesions [*n* = 56]	53/56	49/56	50/56	48/56	50/56	55/56	55/56	55/56
Bone lesions [*n* = 29]^a^	27/29	25/29	25/29	25/29	26/29	23/29	25/29	26/29
All lesions [*n* = 109]	103/109	94/109	94/109	92/109	93/109	101/109	102/109	103/109

All differences are significant with a *p* < 0.01

LN: liver normalization [value divided by hepatic SUVmean]

^a^Two bone metastases newly occurred at T2, and thus, higher values are seen at T2 in all surrogate parameters

## Discussion

As expected, long-term ADT in oligometastatic castration-sensitive patients with prostate cancer resulted in a distinct decrease in the PSA concentration [23]. It could be demonstrated that this PSA response corresponded with the decline in PSMA PET parameters and their derived tumor volumes. However, not all patients responded with their PSA values to the same extent. It is well known that the post-ADT PSA value is of prognostic relevance [24].

Recently, Vaz et al. [14] summarized the currently available clinical (*n* = 9) and in vitro and in vivo (*n* = 10) studies investigating the effect of ADT on PSMA expression. They outlined the high heterogeneity of these 19 reports in terms of study design, numbers of patients or cell lines, hormone sensitivity, ADT type and duration of application. In addition to these heterogeneous study designs, even PSMA expression itself was not measured identically, as it was either measured immunohistochemically or by molecular imaging using PET or SPECT.

Nevertheless, the majority of the collected studies (*n* = 13 reports) indicated increased PSMA expression under ADT in general, including the description of a flare phenomenon [25].

In addition to the castration state, therapy duration appears to be the main reason for the diverging results when interpreting the influence of ADT and PSMA expression. The studies assembled by Vaz et al. [14] suggested that increased PSMA expression under ADT had, in most cases, a maximum therapy span of one month. Additional case reports and small studies supposed a time dependence in which PSMA expression increased under short-term ADT (i.e., 2–6 weeks) and decreased after long-term ADT (i.e., 3–4 months) [26–28].

Afshar-Oromieh et al. [29] reported reduced PSMA expression in PET/CT after long-term (median 230 days) ADT in 10 differently pretreated patients. Recently, Gupta et al. [30] published a lesionwise analysis of 43 therapy-naïve patients with PCa of any stage prior and after a median of 6 months under ADT with the heterogeneous results. The response on ADT measured as PSMA expression differed between the primary tumor and the lymphonodal and bone metastases. While the primary tumor remained visible in all cases, there was complete metabolic remission, especially in oligometastatic disease, in approximately 20% of the lymphonodal and osseous metastases. Nevertheless, even in the primary tumors, the decrease in SUV_max_ correlated with the PSA response. However, a relevant number of both local and distant lesions presented with higher PSMA expression. The PSMA-derived tumor burden for each patient was not analyzed.

In our study with a smaller but homogenous patient group, the analysis of the primary tumor and the metastatic sites prior and after ADT revealed a decrease in PSMA expression in both the primary tumor and metastases, whereby the primary tumor site had the highest PSMA-ligand accumulation both prior and post ADT. However, with decreasing PSMA expression in the primary tumor, the SUV_max_-dependent molecular tumor volumes overestimate the tumor burden due to a lowered tumor-to-background ratio and a resulting blurred tumor delineation.

The strong correlation between the different PET parameters indicated that concordant changes in (molecular) volume and PSMA expression occurred, and the choice of the quantification method appeared secondary. The use of the simply and visually obtainable miPSMA score [19] is practical and did not lead to a clinically relevant loss of information compared to the SUV parameters, not even if separate imaging devices are in use.

Long-term ADT impaired PSMA expression in the vast majority of the primary tumor sites as well as the metastases, resulting in a relevant underestimation of the patient’s tumor burden, especially in the metastatic sites and in lower tumor stages in the majority of patients. Only 47% of the lesions remained detectable under therapy, which corresponds with the results that Afshar-Oromieh et al. [29] observed in their mostly pretreated patient population.

In addition to the overall decrease in PSMA expression, one patient developed two newly detectable bone metastases in our study, while the initial PSMA-positive bone metastases vanished completely under ADT. In other patients, a few metastases showed increased PSMA uptake. These lesions probably indicate, as previously postulated, dedifferentiated [31, 32], castration-resistant cell clones [29] with serious implications for their further therapeutic management [33].

Emmet et al. [34] conducted serial PSMA PET examinations in patients with both castration-sensitive and castration-resistant PCa within 9–28 days after the onset of ADT. They described both a reduction in the SUV_max_ and a positive PSA response in castration-sensitive patients. In castration-resistant individuals, PSMA expression increased, and the PSA response occurred later if at all.

In our study, the PSA response was heterogeneous as well. Nine patients showed a reduced PSA response with PSA values at T2 > 1 ng/ml, which may indicate that hormone resistance is associated with a higher risk of biochemical recurrence [24]. Concomitant with Emmet et al. [34], patients with a sufficient PSA response presented with significantly more irretrievable lesions on T2 PSMA PET. Both patients with complete remission in metabolic imaging at T2 showed a sufficient PSA response. However, one patient in our study with new bone metastases at T2 had a sufficient PSA response at 0.27 ng/ml.

Recently, published studies [35, 36] dealing with biochemically recurrent prostate cancer reported higher tumor detection rates in patients under ADT, suggesting the assumption that the effect of ADT on PSMA expression changes within the course of the disease. As demonstrated in our study and in the study by Gupta et al. [30], ADT masks PSMA expression in early/therapy-naïve stages, and thus, persisting PSMA expression under therapy may be an indicator for early castration resistance, as these lesions are not sufficiently suppressed by ADT and may require further therapeutic approaches. In short, in our therapy-naïve setting under ADT, far fewer lesions could be seen in PSMA PET, as they are not sufficiently controlled by ADT alone.

The limitations of our present study are the low number of patients, the retrospective study design, the heterogeneous ADT and CTx and, of course, the lack of histologic confirmation of the lesion’s malignancy in follow-up.

## Conclusion

The detectability of both the primary tumor and the metastases in lymph nodes and bone in PSMA PET decreased early after the onset of ADT, especially in patients with a sufficient PSA response (PSA at T2 < 1 ng/ml). PSMA PET acquired after initiation of ADT (> 4–6 weeks) led to an underestimation of the miTNM stage in the majority of patients.

There might be greater potential in the post-ADT (T2) PET than widely supposed, as these fewer lesions might be those that cannot be controlled by ADT alone and thus might require different treatment strategies, e.g., molecular image guided local ablative therapy. Furthermore, prospective research is necessary to evaluate the potential benefit of that approach.

## Data Availability

Anonymized data can be offered upon request.

## Abbreviations

ADT: Androgen deprivation therapy
CT: Computed tomography
^[68^Ga]Ga-PSMA-11: [^68^Ga]Ga-prostate-specific membrane antigen 11
FOLH1: Folate hydrolase 1
GSC: Gleason score
miPSMA Score: Molecular imaging prostate-specific membrane antigen score
MRI: Magnetic resonance imaging
OSEM: Ordered subset expectation maximization
PCa: Prostate cancer
PET: Positron emission tomography
PSA: Prostate-specific antigen
PSMA: Prostate-specific membrane antigen
PSMA-TV: Prostate-specific membrane antigen-derived tumor volume
SPECT: Single-photon emission computed tomography
SUV_max_: Maximum standard uptake value
SUV_mean_: Mean standard uptake value
TL-PSMA: Total lesion prostate-specific membrane antigen
TOF: Time-of-flight
VOI: Volumes of interest
